# Influences of Plasma Plume Length on Structural, Optical and Dye Degradation Properties of Citrate-Stabilized Silver Nanoparticles Synthesized by Plasma-Assisted Reduction

**DOI:** 10.3390/nano12142367

**Published:** 2022-07-11

**Authors:** Tirtha Raj Acharya, Geon Joon Lee, Eun Ha Choi

**Affiliations:** 1 Department of Electrical and Biological Physics, Kwangwoon University, Seoul 01897, Korea; tirtharajacharya2050@gmail.com; 2 Plasma Bioscience Research Center, Kwangwoon University, Seoul 01897, Korea

**Keywords:** silver nanoparticles, plasmon, plasma synthesis, plasma-treated water, reactive oxygen species, citrate

## Abstract

Citrate-capped silver nanoparticles (*Ag@Cit NPs*) were synthesized by a simple plasma-assisted reduction method. Homogenous colloidal *Ag@Cit NPs* solutions were produced by treating a $AgNO_{3}$-trisodium citrate-deionized water with an atmospheric-pressure argon plasma jet. The plasma-synthesized *Ag@Cit NPs* exhibited quasi-spherical shape with an average particle diameter of about 5.9−7.5 nm, and their absorption spectra showed surface plasmon resonance peaks at approximately 406 nm. The amount of *Ag@Cit NPs* increased in a plasma exposure duration-dependent manner. Plasma synthesis of *Ag@Cit NPs* was more effective in the 8.5 cm plume jet than in the shorter and longer plume jets. A larger amount of *Ag@Cit NPs* were produced from the 8.5 cm plume jet with a higher pH and a larger number of aqua electrons, indicating that the synergetic effect between plasma electrons and citrate plays an important role in the plasma synthesis of *Ag@Cit NPs*. Plasma-assisted citrate reduction facilitates the synthesis of *Ag@Cit NPs*, and citrate-capped nanoparticles are stabilized in an aqueous solution due to their repulsive force. Next, we demonstrated that plasma-synthesized *Ag@Cit NPs* exhibited a significant degradation of methylene blue dye.

## 1. Introduction

Metal nanoparticles have interesting physical, electrical, optical, and antimicrobial characteristics that make them useful in various fields, including biomedicine, healthcare, food storage, textiles, agriculture, catalysis, optical sensors, flexible electronics, solar cells, drug delivery, cosmetics, antimicrobial action, chemical sensing, and environmental engineering [1,2,3]. Silver nanoparticles (*AgNPs*) can be fabricated by various methods, such as thermal evaporation [4], plasma synthesis [5,6,7], microwave-assisted synthesis [8], gamma ray-assisted synthesis [9], spray pyrolysis [10], laser ablation [11], citrate reduction [12,13,14], and electrochemical synthesis [15]. Among these synthesis routes, the chemical reduction method has been widely used due to its advantages of simplicity, high yield, no aggregation, and low cost [7,12,13]. To create silver nanoparticles, it is necessary to add chemical reducing agents to silver salt ions and reduce silver ions in boiling water. In addition, stabilizers such as citrate [16], polyvinyl alcohol [17], polyvinyl pyrrolidone [6], bovine serum albumin [18], and cellulose [19] are extensively used to avoid aggregation of nanoparticles.

Recently, atmospheric-pressure plasma jets have received a lot of interest as a promising green means for the synthesis of nanomaterials [5,20]. Metallic nanoparticles can be produced by applying atmospheric-pressure plasma jets [5,20], dielectric barrier discharge plasma [21], gas–liquid interfacial plasmas [22], and dual plasma electrolysis [23] to aqueous metal salt solutions. Among these plasma synthesis methods, metal nanoparticle synthesis with atmospheric-pressure plasma jets is relatively simple, cost-effective, and efficient. Interestingly, plasma synthesis of metal nanoparticles can be carried out in a shorter processing time as compared with their synthesis by the chemical reduction method. Furthermore, the plasma jets can synthesize metal nanoparticles in an atmospheric environment without vacuum devices, and can be operated using various gases. Noble gases such as argon, helium, and neon are easily ionized as compared to air [24]. In this research, argon gas was used as the plasma working gas. Argon is the cheapest noble gas. The Ar plasma jets are simple, affordable, and easy to design. The plasma jets can produce reactive oxygen and nitrogen species, charged particles, ultraviolet (UV) light, and electric fields. When the plasma jet enters the deionized water (DW), additional reactive species are generated by plasma–liquid interactions [25,26,27]. If the plasma jet is applied to an aqueous metal salt solution, plasma-generated reactive species will be affected by the reducing and scavenging activities of the plasma-activated medium [5,25,26]. Plasma-assisted reduction of metal ions can be employed for the synthesis of various metal nanoparticles, such as Ag, Au, Cu, Fe, and alloy nanoparticles [5,20,28]. In the plasma-assisted reduction of metal ions, plasma-induced reactive species determine key parameters affecting the synthesis of metal nanoparticles. The amount of plasma-synthesized metal nanoparticles depends on the aqua electron density and the acidity of the plasma-activated medium [5,24]. Kondeti et al. synthesized silver nanoparticles using a hydrogen plasma jet [5], and Lee et al. produced silver nanoparticles using liquid phase plasma [7]. However, no systematic study has been performed on the plasma synthesis mechanism of silver nanoparticles by atmospheric-pressure argon plasma jets and the influence of plasma plume length on the structural and optical properties of plasma-synthesized silver nanoparticles.

In this research, we used the plasma-assisted reduction method to synthesize citrate-stabilized silver nanoparticles (*Ag@Cit NPs*). An atmospheric-pressure argon plasma jet was used for plasma synthesis of *Ag@Cit NPs* in a single-step approach. Trisodium citrate (TSC) was used to stabilize *AgNPs* and to enhance the plasma synthesis efficiency. To diagnose the plasma, key plasma parameters such as current–voltage waveforms of plasma discharge, electron density, electron temperature, and optical emission spectra of the plasma plume, were measured. To study the effects of plasma treatment on DW and $AgNO_{3}$-TSC-DW, their acidity and electrical conductivity were measured. To analyze the structural and optical properties of plasma-synthesized *Ag@Cit NPs*, their transmission electron microscopy (TEM) images and optical absorption spectra were examined. To identify plasma synthesis routes of *Ag@Cit NPs*, the effects of plasma treatment duration and plasma plume lengths on plasma-synthesized *Ag@Cit NPs* were investigated. To demonstrate applications of plasma-synthesized *Ag@Cit NPs*, the degradation effects of plasma-synthesized *Ag@Cit NPs* on methylene blue (MB) dye were measured.

## 2. Experimental Methods

### 2.1. Characteristics of the Plasma Device

**Figure 1:**
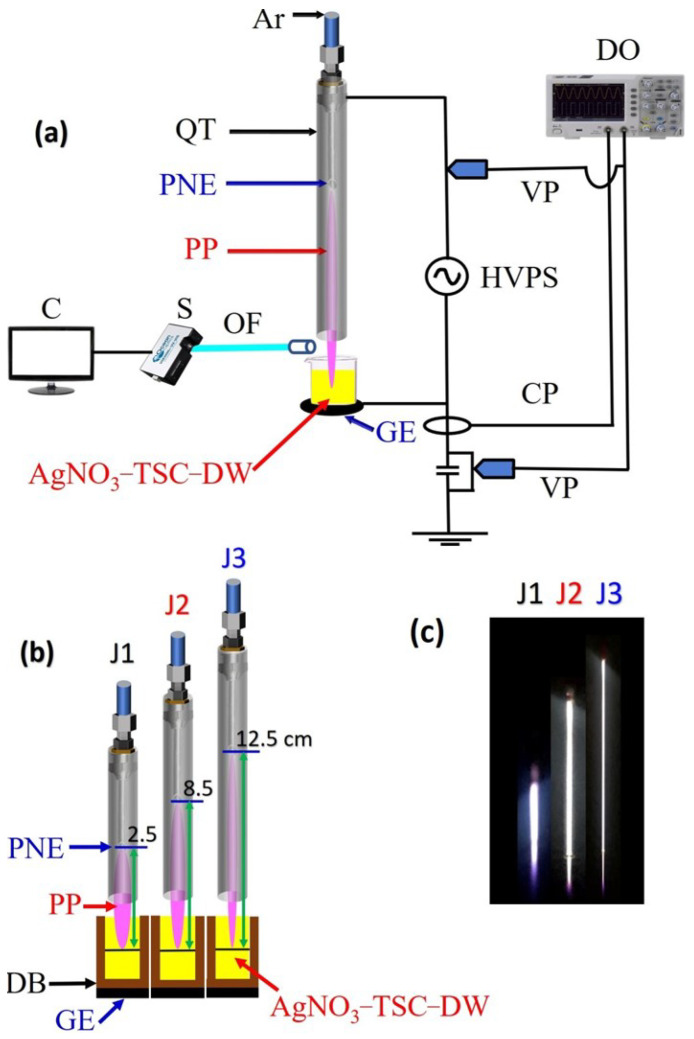
(**a**) Experimental layout of a plasma jet device for synthesis of silver nanoparticles; (**b**) schematic diagram; (**c**) photos of the J1, J2, and J3 jets. In (**a**,**b**), Ar, argon gas; QT, quartz tube; PNE, powered needle electrode; PP, plasma plume; OF optical fiber; S, spectrometer; C, computer; **DB**, dielectric barrier; GE, grounded electrode; ${\mathrm{AgNO}}_{3}\text{-}\mathrm{TSC}\text{-}\mathrm{DW}$, $AgNO_{3}$-TSC-DW solution for synthesis of silver nanoparticles; HVPS, high voltage power supply; VP, voltage probe; CP, Current probe; DO, digital oscilloscope.

In this research, an alternating current (AC)-driven argon plasma jet (Figure 1) was used for synthesis of *Ag@Cit NPs*. The plasma device consists of a powered needle electrode and a grounded copper tape electrode. A stainless-steel needle electrode (with a length of 50 mm, an inner diameter of 0.6 mm, and an outer diameter of 0.9 mm) was used as the power electrode. For grounding, a copper tape was fixed to the bottom of a glass beaker that contained $AgNO_{3}$-TSC-DW. A plasma jet was created from a needle electrode inserted inside a quartz tube (with an inner diameter of 2 mm and an outer diameter of 4 mm). Argon gas entered the quartz tube through a needle, and was supplied to the needle with a gas flow rate of 500 standard cubic centimeters per minute (sccm). As plasma can propagate a long distance along an argon gas stream in a quartz tube, plasma plume length is longer for a longer quartz tube [29]. Three different plasma jets with plume lengths of 2.5, 8.5, and 12.5 cm were used to study the effect of plasma plume length on the synthesis of *Ag@Cit NPs*. To make plasma jets with plume lengths of 2.5, 8.5, and 12.5 cm, a powered needle electrode was inserted inside each of 6.5, 11.5, and 15.5 cm long quartz tubes (Figure 1b). In this research, plasma jets with plume lengths of 2.5, 8.5, and 12.5cm are called J1, J2, and J3 jets, respectively, (Figure 1c). When the plasma jet exited the nozzle of the quartz tube, the plasma plume region was at a lower pressure than the surrounding ambient air. Thus, the ambient air diffused into and directly mixed with the plasma plume below the nozzle of the quartz tube. Due to the quenching effect of atmospheric air, the plasma plume was weakened after traveling a few centimeters away from the nozzle of the quartz tube. The plasma plume lengths of J1, J2, and J3 jets were longer than their needle-to-nozzle distances. After the plasma jet exited the nozzle of the quartz tube, it propagated a few centimeters away from the nozzle. In Figure 1c, the purple color at the end of the plasma plume was due to the nitrogen emission obtained from the diffused air. After nitrogen emission, the plasma plume dissipated. In this research, the plasma jet entered the aqueous silver salt solution, and the penetration depth of the plasma plume from the water surface was adjusted to be approximately 5 mm. As the plasma plume was charged and the end of it was in contact with the water–air interface, the electric field was applied to aqua electrons and silver ions in the solutions.

To investigate the reactive oxygen and nitrogen species produced by the plasma jet, optical emission spectra were recorded by a fiber optic spectrometer (HR4000, Ocean Optics, Orlando, FL, USA). Optical emission of the propagating plasma jet was collected near the water–air interface, as shown in Figure 1a. Meanwhile, Balmer emission of excited hydrogen atoms was measured in the end-on geometry to detect hydrogen produced in the $AgNO_{3}$-TSC-DW by the plasma-liquid interactions. For this purpose, a copper tape with a circular hole was fixed to the bottom of a beaker that contained $AgNO_{3}$-TSC-DW, and hydrogen emission was measured from the plasma light that transmitted through a hole of a copper electrode below the plasma-treated $AgNO_{3}$-TSC-DW. To examine surface plasmon resonance effects, absorption spectra of plasma-synthesized *Ag@Cit NPs* were measured by an optical absorption spectrometer (Maya2000Pro, Ocean Insight, Orlando, FL, USA). The pH and electrical conductivity of the plasma-treated-$AgNO_{3}$-TSC-DW were measured by pH and conductivity meters (${\mathrm{PCTSTestr}}^{\mathrm{TM}}\;50$, Oakton, Melbourne, Australia).

### 2.2. Electrical Characteristics of Plasma Discharge

**Figure 2:**
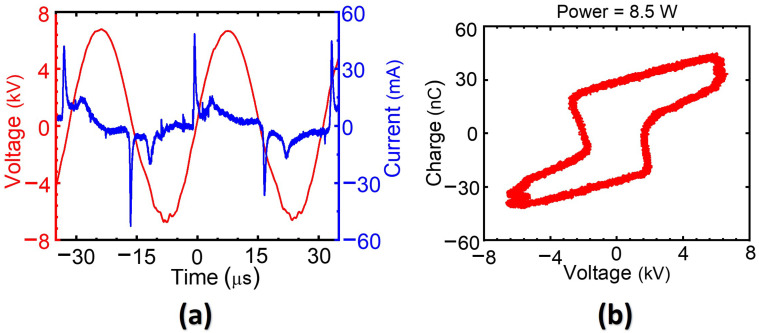
(**a**) Typical current and voltage waveforms of the plasma discharge for the J2 jet, and (**b**) corresponding Lissajous plot.

To measure the applied voltage and discharge current, a high-voltage probe (P6015A, Tektronix, Beaverton, OR, USA) and a current probe (CP030, LeCroy, Chestnut Ridge, NY, USA) were connected to a digital oscilloscope (WaveSurfer 434, Lecroy, New York, NY, USA). Figure 2a shows typical current and voltage waveforms of the AC-driven plasma discharge for the J2 jet. Several positive current peaks appeared with increasing applied voltage and resulted in accumulation of charges inside the quartz tube. The accumulation of positive surface charges in the positive half-cycle of the applied voltage caused discharge current peaks, and the polarity of these charges was reversed in the negative half-cycle of the applied voltage. The measured discharge currents of J1, J2, and J3 jets were 46, 43, and 34 mA, respectively, at a fixed peak voltage of 4.8 kV (rms). The current and voltage waveforms had a period of 31.5 μs, which corresponded to a frequency of 32 kHz. To determine the accumulated charge, we connected a 10 nF ceramic capacitor in series to the ground electrode and measured the voltage across it. The corresponding charge (Q)–voltage (V) Lissajous plot is shown in Figure 2b. The Q–V plot forms a closed loop, and its area represents the total energy dissipated in one cycle [30]. The dissipated powers of J1, J2, and J3 jets were measured to be 8.9, 8.5, and 4.0 W, respectively, corresponding to unit cycle energy dissipation of 0.28, 0.27, and 0.13 mJ.

**Figure 3:**
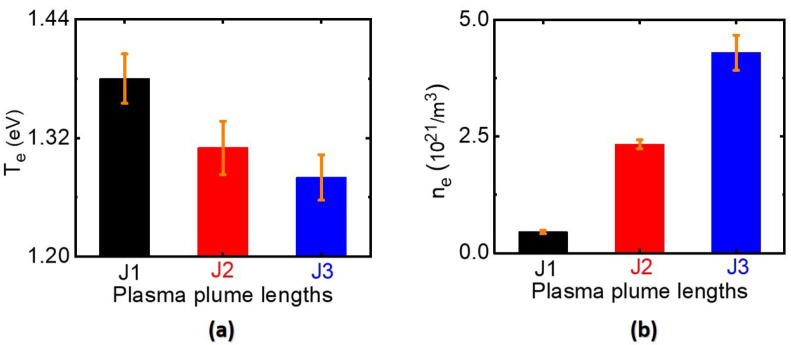
(**a**) Electron temperature and (**b**) electron density of the J1, J2, and J3 jets.

Electron temperature and density are key plasma parameters affecting the synthesis of metal nanoparticles [25,31,32]. Energy transfer from the thermally excited electrons to the ions increased due to the high electron densities in the discharged plasma, and the electron cooling rate was high. Higher electron densities typically imply lower electron temperatures, and vice versa. Long plasma jets feature more frequent elastic collisions between electrons and heavier particles than do shorter plasma jets. Consecutive energy impact collisions with heavy particles cooled the electron temperature of the long jet rather than that of the short jet. Electron temperature and density also have an impact on the interaction between the plasma jet and the liquid medium [26,31]. In the plasma-assisted reduction of silver ions, the amount of plasma-synthesized silver nanoparticles depends on the electron density and acidity of plasma-activated medium. Electron temperatures and densities of the J1, J2, and J3 jets at the nozzle of the quartz tube are shown in Figure 3a,b. Electron temperatures of the J1, J2, and J3 jets were 1.38, 1.31, and 1.28 eV, respectively. Electron densities of J1, J2, and J3 jets were $0.45\times 10^{21}$, $2.33\times 10^{21}$, and $4.30\times 10^{21}$ electrons/m${}^{3}$, respectively. The collisional radiative model [33] and the convective wave packet model [34] were used to compute electron temperature and density, respectively.

### 2.3. Plasma Synthesis of Silver Nanoparticles

Silver nitrate ($AgNO_{3}$) and trisodium citrate (TSC, ${\mathrm{Na}}_{3}C_{6}H_{5}O_{7}$) were purchased from Sigma-Aldrich (Yongin-city, Kyunggi-do, Korea). Stock solutions of 25 mM $AgNO_{3}$ and 50 mM TSC were prepared. To synthesize *Ag@Cit NPs*, solutions of 4.0 mM $AgNO_{3}$ and 8.0 mM TSC ($AgNO_{3}$:TSC = 1:2) were prepared by mixing 4.8 mL of 25 mM $AgNO_{3}$ and 4.8 mL of 50 mM TSC with 20 mL DW, respectively. Then, 8 mL of the $AgNO_{3}$-TSC-DW was treated by the atmospheric-pressure Ar plasma jet [5,16]. The plasma treatment durations were 2, 5, 7.5, and 10 min. To find the optimum conditions for plasma synthesis and to study plasma synthesis routes, we investigated the effects of plasma treatment duration and plasma plume length on the physical properties of plasma-synthesized *Ag@Cit NPs*.

### 2.4. Structural and Optical Characterizations of Plasma-Synthesized Silver Nanoparticles

To investigate the structural properties of plasma-synthesized *Ag@Cit NPs*, high-resolution transmission electron microscopy (HR-TEM) and EDS mapping were conducted using a Cs-corrected scanning transmission electron microscope (Neo ARM, JEOL, Tokyo, Japan). For measuring TEM images, the *Ag@Cit NPs* solution was drop-cast on a carbon-stabilized Formvar-coated 200 mesh copper TEM grid (Ted Pella #01801, Redding, CA, USA), and dried at room temperature under atmospheric conditions. The size distributions of plasma-synthesized *AgNPs* were obtained by analysis with ImageJ software, v1.53s (National Institutes of Health, Bethesda, MD, USA). To confirm the surface plasmon resonance effects, absorption spectra of plasma-synthesized *Ag@Cit NPs* were measured by an optical absorption spectrometer (Maya2000Pro, Ocean Insight, Orlando, FL, USA).

### 2.5. Measurements of Dye Degradation by Plasma-Synthesized Silver Nanoparticles

A nonbiodegradable and commonly used organic dye, methylene blue (MB), was selected as a model analyte. MB and sodium borohydride ($NaBH_{4}$) were purchased from Sigma-Aldrich (Yongin-city, Kyunggi-do, Korea). To investigate the degradation activity of MB dye by plasma-synthesized *Ag@Cit NPs*, 0.3 mL of 1 mM MB was mixed with 10 mL DW, 0.3 mL of 100 mM ${\mathrm{NaBH}}_{4}$, and 1.0 mL of *Ag@Cit NPs*. For the dye degradation experiment, the plasma-synthesized *Ag@Cit NP* solutions were diluted 11.6-fold with DW. Consequently, solutions with 26 μM MB were treated by the plasma-synthesized *Ag@Cit NPs*. The *Ag@Cit NP* treatment duration was 10 min. Optical absorption spectra of *Ag@Cit NP*-treated and -untreated MB solutions were measured by an optical absorption spectrometer (Maya2000Pro, Ocean Insight, Orlando, FL, USA).

Next, we investigated dye degradation activity of plasma-synthesized *Ag@Cit NPs* coated onto filter paper to achieve dye degradation without remnant silver nanoparticles. The filter paper was immersed in a *Ag@Cit NP* solution for 3 h and dried at 60 ${}^{{}^{\circ}}$C for 1 h. After drying, most of the plasma-activated liquid was removed. In this research, *Ag@Cit NPs* loaded onto the filter paper is called *Ag@Cit NP*-paper. To examine dye degradation effects of the *Ag@Cit NP*-paper, *Ag@Cit NP*-coated and -uncoated papers were soaked in 26 μM MB solutions. After dye degradation treatment for 1h in the *Ag@Cit NP*-paper-containing solution, pristine and *Ag@Cit NP*-paper-treated MB solutions were transferred into a 15 mL conical tube and used for further experiments.

## 3. Results and Discussion

### 3.1. Optical Properties of the Plasma Jet

**Figure 4:**
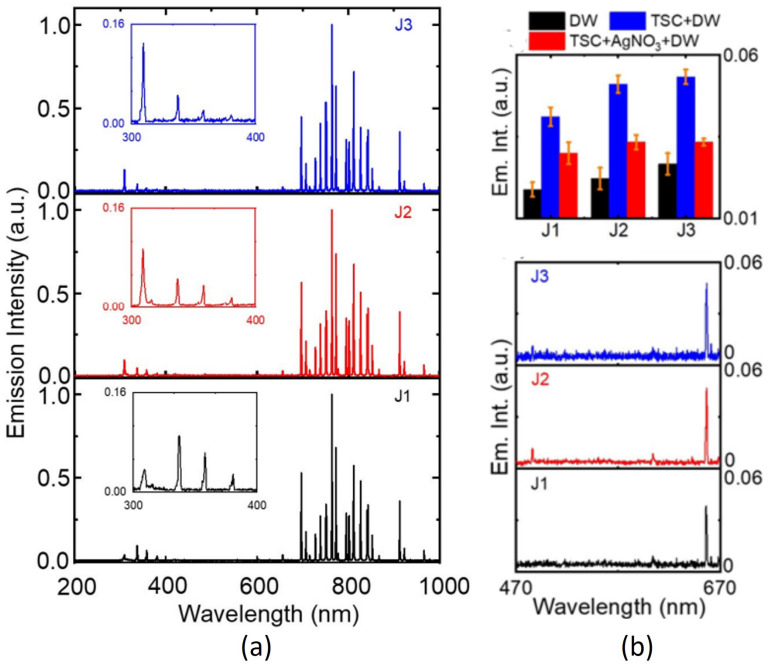
(**a**) Optical emission spectra of the J1, J2, and J3 jets in the 200−1000 nm range. The 309 nm and 330–380 nm emission bands correspond to the hydroxyl radical and the second positive system transition of nitrogen molecules, respectively. (**b**) (**top**) Hydrogen emission intensities of DW, TSC-DW, and $AgNO_{3}$-TSC-DW treated for 10 min with the J1, J2, and J3 jets. (**bottom**) Hydrogen emission spectra of $AgNO_{3}$-TSC-DW treated for 10 min with the J1, J2, and J3 jets.

Optical emission spectroscopy is a common, fast, simple, and straightforward method to investigate the various reactive and energized species in the plasma jet. Optical emission spectra (OES) of the J1, J2, and J3 jets in the 200–1000 nm region displayed the characteristic emission peaks of plasma-induced reactive oxygen and nitrogen species, as shown in Figure 4a. The emission lines in the 700–900 nm region are attributed to 2p→1s transition of excited argon molecules [35]. Intense emission at 309 nm corresponds to the hydroxyl radical (OH) produced by breakdown of water molecules present in ambient air under the influence of electrons and metastable atoms [36,37]. At the plasma–liquid interface, prolonged plasma exposure causes a dissociation of water molecules by energetic ions from the plasma jet, resulting in OH radical generation. Weak emission bands in the 330−380 nm range are generated from the second positive system transition of nitrogen molecules ($C^{3}\Pi_{u}\to B^{3}\Pi_{g}$) [37,38], which can be ascribed to plasma operation under atmospheric conditions. The emission at 777 nm was attributed to $2s^{2}2p^{3}{(}^{4}S^{0})3s\to 2s^{2}2p^{3}{(}^{4}S^{0})3p$ transition of atomic oxygen (*O*) [39]. The emission line at 656 nm was attributed to Balmer $H_{\alpha }$ transition of excited hydrogen atoms (Figure 4b). The Balmer emission indicates that hydrogen atoms were produced in the plasma-synthesized $AgNO_{3}$-TSC-DW. Under our experimental conditions, most of the hydrogen atoms were produced from the plasma electron reduction of hydrogen ions that came from citrate (TSC). As shown in Figure 4b, the hydrogen emission intensity was stronger in the J2 and J3 jets than the J1 jet, which was attributed to the higher concentration of plasma electrons in the longer jet. Meanwhile, the hydrogen emission intensity of the J3 jet was lower than the expectation from the plasma electron-induced reduction of hydrogen ions. The hydrogen emission intensity of the J3 jet was slightly higher than that of the J2 jet. Hydrogen generation by plasma electron reduction of hydrogen ions may compete with other product generation processes by reaction of hydrogen atoms with plasma-induced reactive species (hydroxyl radical, hydroperoxyl radical, etc.). Next, the J1 jet produced more reactive nitrogen species (RNS) than the longer jet (Figure 4a). Only a small fraction of nitrogen molecules can be dissociated in the outflow of the longer jet due to the lower electron temperature. Electron excitation and ionization of nitrogen molecules are more favorable in the J1 jet because the shorter jet exhibited a higher electron temperature than the longer jet (Figure 3). The binding of plasma-induced nitrate (nitrite) with hydrogen ions led to acidic water and prevents $H^{+}$–to–H conversion, leading to the suppression of the plasma synthesis of *AgNPs* in the J1 jet. Overall, the longer jet generated a larger number of hydrogen atoms and a smaller amount of RNS in the $AgNO_{3}$-TSC-DW.

### 3.2. Electrical Conductivity and Acidity of Plasma-Treated Silver Precursor Solutions

**Figure 5:**
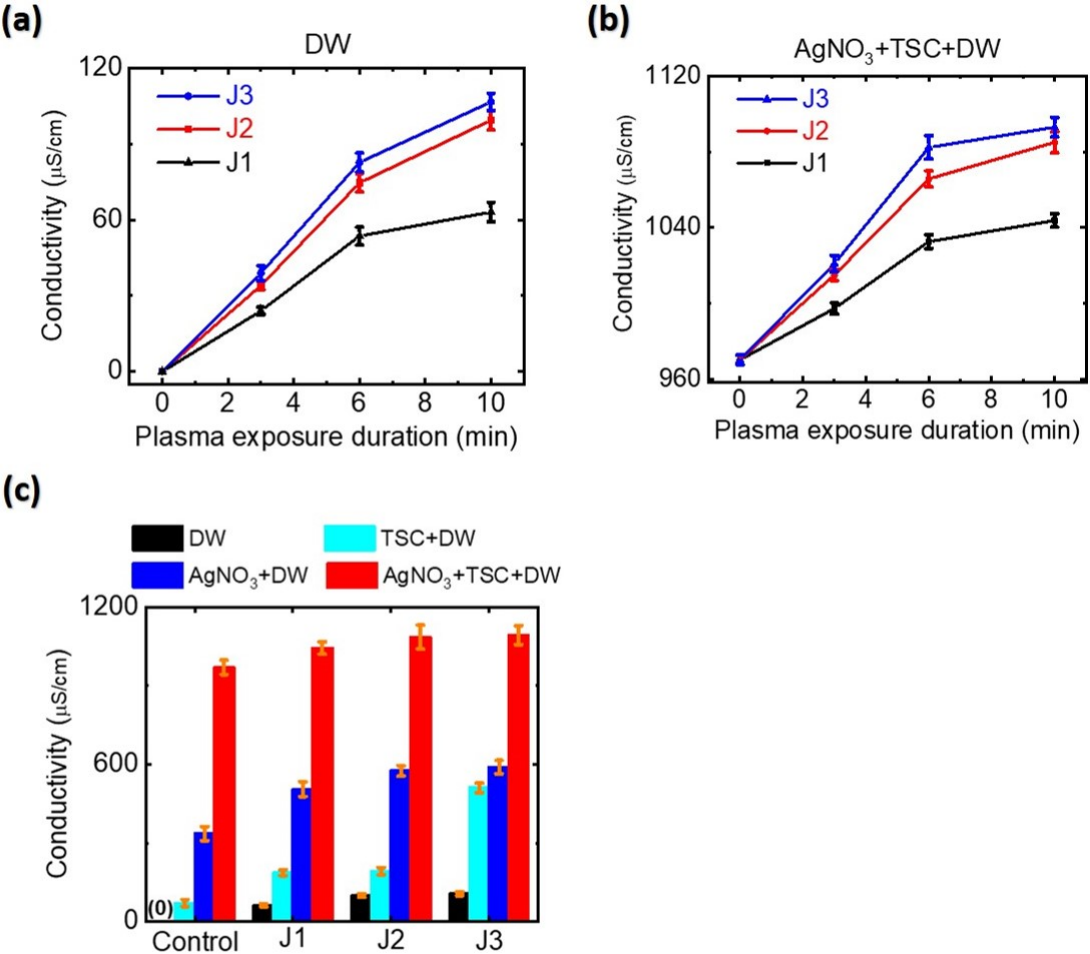
Electrical conductivies of (**a**) plasma-treated-DW and (**b**) plasma-treated $AgNO_{3}$-TSC-DW as a function of plasma exposure duration. (**c**) Electrical conductivies of DW, TSC-DW, $AgNO_{3}$-DW, and $AgNO_{3}$-TSC-DW treated for 10 min with the J1, J2, and J3 jets. In Figure (**c**), the electrical conductivity of plasma-untreated DW was 0 μS/cm.

**Figure 6:**
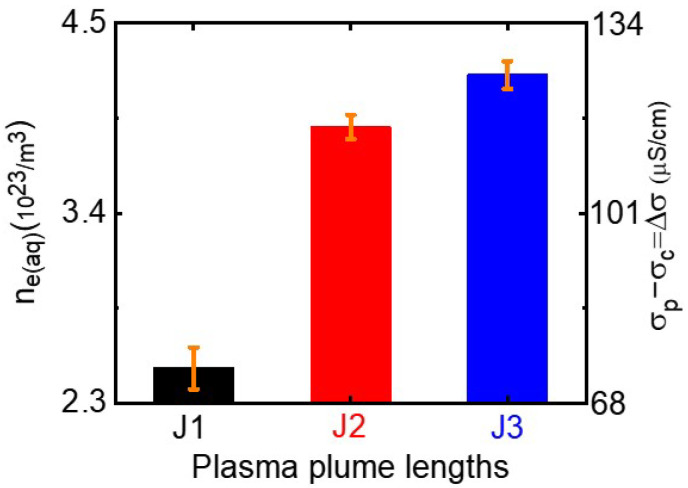
Aqua electron densities of plasma-treated $AgNO_{3}$-TSC-DW treated for 10 min with the J1, J2, and J3 jets.

Electrical conductivity and pH are key parameters governing the physicochemical properties of a plasma-activated medium. Atmospheric-pressure plasma in contact with an aqueous solution produces aqua electrons, resulting in the transport of electrons from the gaseous plasma into the aqueous solution [40,41]. Aqua electrons can affect the electrical conductivity and acidity of plasma-treated silver precursor solution. To study the plasma synthesis mechanism of *AgNPs*, we measured the conductivies and pH values of plasma-treated silver precursor solutions. As shown in Figure 5, the electrical conductivies of the plasma-treated-DW and $AgNO_{3}$-TSC-DW increased significantly with plasma treatment duration. When DW was treated with the plasma jet for 10 min, the electrical conductivies of plasma-treated-DW rose from 0 μS/cm to 63, 99, and 107 μS/cm for the J1, J2, and J3 jets, respectively (Figure 5a). The electrical conductivies of plasma-treated $AgNO_{3}$-TSC-DW jumped from 970 μS/cm to 1044, 1085, and 1093 μS/cm for the J1, J2, and J3 jets, respectively, (Figure 5b). The plasma-untreated $AgNO_{3}$-TSC-DW showed electrical conductivity of 970 μS/cm. During plasma treatment, plasma-generated aqua electrons accumulated in the plasma-treated $AgNO_{3}$-TSC-DW. As shown in Figure 5c, $AgNO_{3}$ and TSC contribute to the electrical conductivity of the $AgNO_{3}$-TSC-DW. Plasma treatment for $AgNO_{3}$-DW, TSC-DW, and $AgNO_{3}$-TSC-DW increased their electrical conductivies due to plasma-generated aqua electrons. By subtracting the electrical conductivity of plasma-untreated $AgNO_{3}$-TSC-DW ($\sigma_{c}$) from the electrical conductivity of the plasma-treated $AgNO_{3}$-TSC-DW ($\sigma_{p}$), we obtained the electrical conductivity of plasma-generated aqua electrons ($\sigma_{e(aq)}=\sigma_{p}-\sigma_{c}$). For a plasma treatment duration of 10 min, the electrical conductivities of plasma-generated aqua electrons were estimated to be 74, 115, and 123 μS/cm for the J1, J2, and J3 jets, respectively, as shown in Figure 6. The concentration of plasma-induced aqua electrons can be estimated from their electrical conductivity: $n_{e(aq)}=\sigma_{e(aq)}/\left(e\cdot \mu_{e}\right)$. Here, $n_{e(aq)}$, e, and $\mu_{e}$ are the concentration, charge, and mobility of aqua electrons in the plasma-treated-$AgNO_{3}$-TSC-DW, respectively. From the reported value ($\mu_{e}=1.84\times 10^{-3}{\mathrm{cm}}^{2}/\left(V\cdot s\right)$) for the mobility of aqua electrons [42], we can estimate the aqua electron density of plasma-activated medium. For a plasma treatment duration of 10 min, the aqua electron densities of plasma-treated $AgNO_{3}$-TSC-DW increased to 2.5 × $10^{21}$/$m^{3}$, 3.9 × $10^{21}/m^{3}$, and 4.2 × $10^{21}/m^{3}$ for the J1, J2 and J3 jets, respectively, as shown in Figure 6. The aqua electron density of the longer jet was higher than that of the shorter jet.

**Figure 7:**
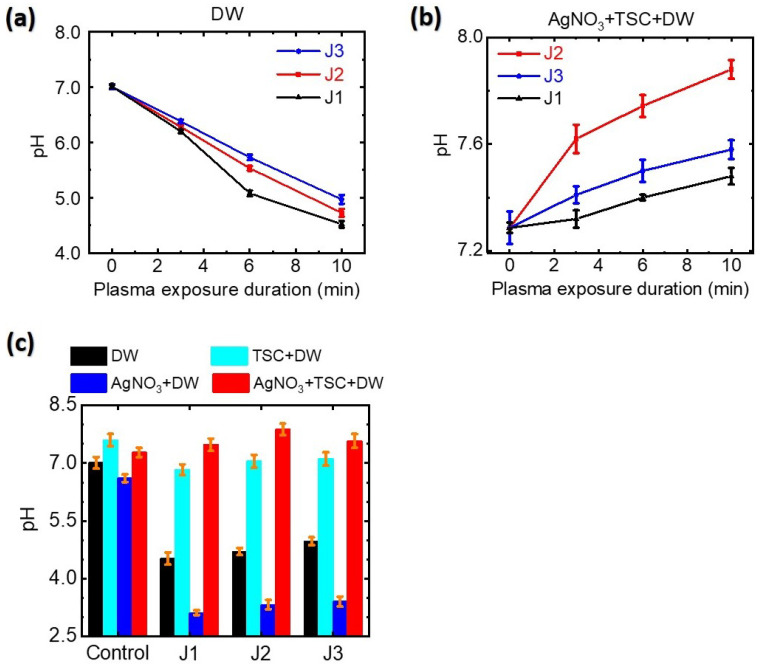
pH values of (**a**) plasma-treated-DW and (**b**) plasma-treated $AgNO_{3}$-TSC-DW as a function of plasma exposure duration. (**c**) pH values of DW, TSC-DW, $AgNO_{3}$-DW, and $AgNO_{3}$-TSC-DW treated for 10 min with the J1, J2, and J3 jets.

When DW was treated for 10 min with the plasma jets, the pH values of plasma-treated-DW dropped from 7.0 to 4.5, 4.7, and 5.0 for the J1, J2, and J3 jets, respectively, as shown in Figure 7a. Prolonged plasma treatment of DW produced a lower pH. Plasma treatment of DW produced acidic water due to the plasma-induced hydrogen ion–nitrate (nitrite) complex formation. When TSC-DW was treated with the plasma jets for 10 min, the pH values of plasma-treated-TSC-DW were 6.8, 7.1, and 7.2 for the J1, J2, and J3 jets, respectively, (Figure 7c). The pH values of plasma-treated-TSC-DW are higher than those of plasma-treated-DW, indicating that TSC can scavenge plasma-generated hydrogen ions. For a plasma exposure duration of 10 min, the pH values of plasma-treated $AgNO_{3}$-TSC-DW increased slightly to 7.3 to 7.5, 7.9, and 7.6 for the J1, J2, and J3 jets, respectively (Figure 7b). As described in the following section, plasma-assisted citrate reduction of silver ions can be facilitated at higher pH values.

### 3.3. Structural and Optical Properties of Plasma-Synthesized Silver Nanoparticles

**Figure 8:**
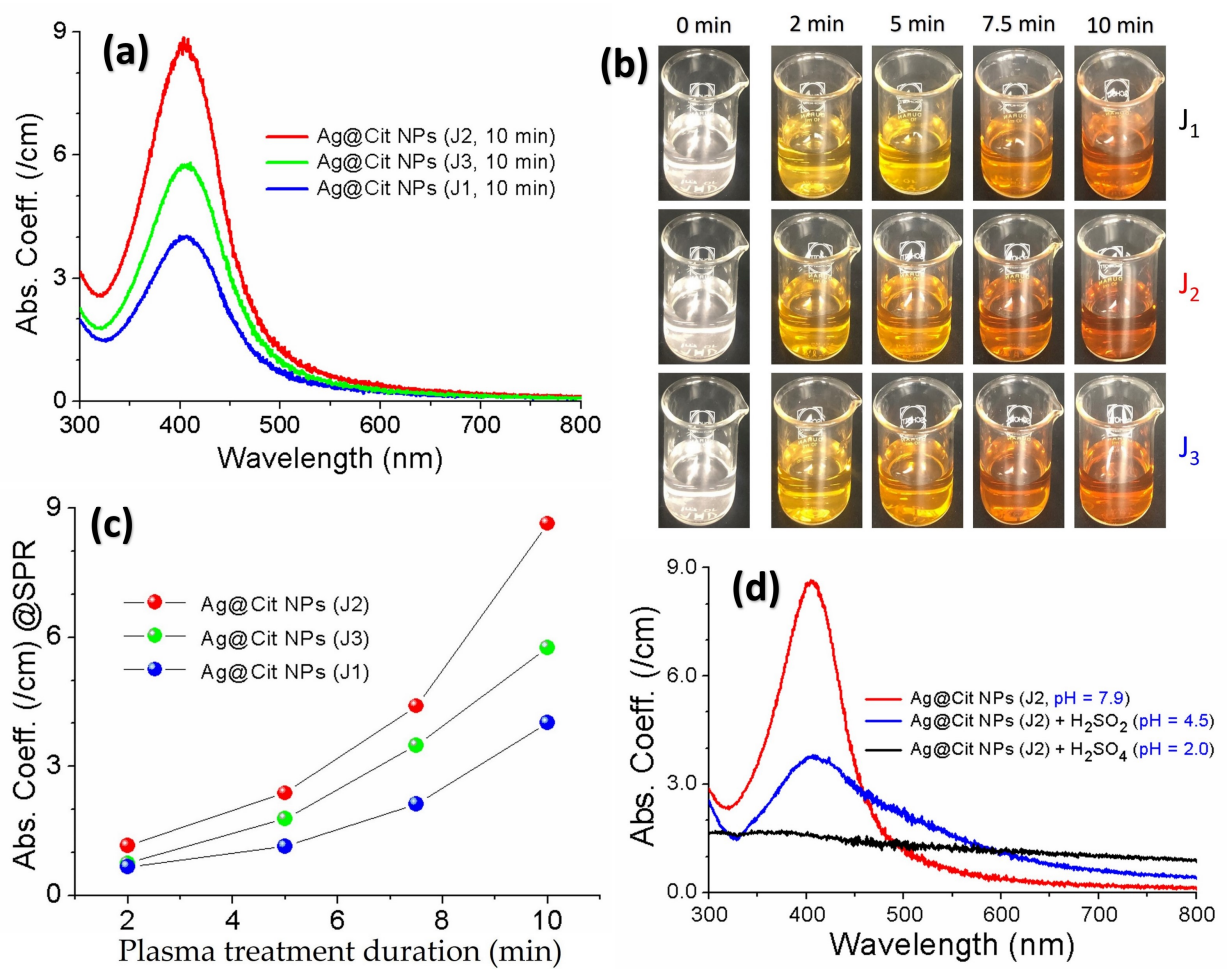
(**a**) Optical absorption spectra of the *Ag@Cit NPs* synthesized by treating $AgNO_{3}$-TSC-DW for 10 min with the J1, J2, and J3 jets. (**b**) Color change of the plasma-synthesized *Ag@Cit NPs* over the plasma treatment durations of 2, 5, 7.5, and 10 min. (**c**) SPR peak intensity of the *Ag@Cit NPs* as a function of the plasma treatment duration. (**d**) Effects of acid treatments on the surface plasmon resonance absorption band of the *Ag@Cit NPs* synthesized with the J2 jet: $H_{2}SO_{4}$ (pH = 2) and $H_{2}SO_{4}$ (pH = 4.5).

After the $AgNO_{3}$-TSC-DW was treated with the plasma jet for a few minutes, the colors of the silver precursor solutions changed from colorless to yellow (Figure 8b), indicating that *Ag@Cit NPs* were successfully generated by plasma-assisted citrate reduction. Absorption spectra of the plasma-synthesized *Ag@Cit NPs* exhibited surface plasmon resonance (SPR) bands at approximately 406 nm (Figure 8a). As the plasma treatment duration increased from 2 min to 10 min, absorption coefficients of *Ag@Cit NP* solutions at the SPR peaks increased from 0.67/cm, 1.2/cm, and 0.75/cm to 4.0/cm, 8.6/cm, and 5.8/cm for the J1, J2 and J3 jets, respectively, (Figure 8c), indicating that prolonged plasma treatment produced a larger amount of *Ag@Cit NPs*. This happened because prolonged plasma treatment of silver precursor solutions led to more plasma reductants to be used for synthesis of *Ag@Cit NPs*. Regarding the effect of plasma plume length on the synthesis of *Ag@Cit NPs*, the SPR peak intensity of *Ag@Cit NPs* was stronger in the J2 jet than the J1 and J3 jets, indicating that the J2 jet can synthesize a larger amount of *Ag@Cit NPs* than the shorter and longer jets. For a plasma treatment duration of 10 min, the SPR wavelengths of *Ag@Cit NPs* were 405.9, 405.0, and 406.9 nm for the J1, J2, and J3 jets, respectively, indicating similar average particle sizes of plasma-synthesized *Ag@Cit NPs*. This is because TSC stabilizes *Ag@Cit NPs* in an aqueous solution by suppressing their growth.

**Figure 9:**
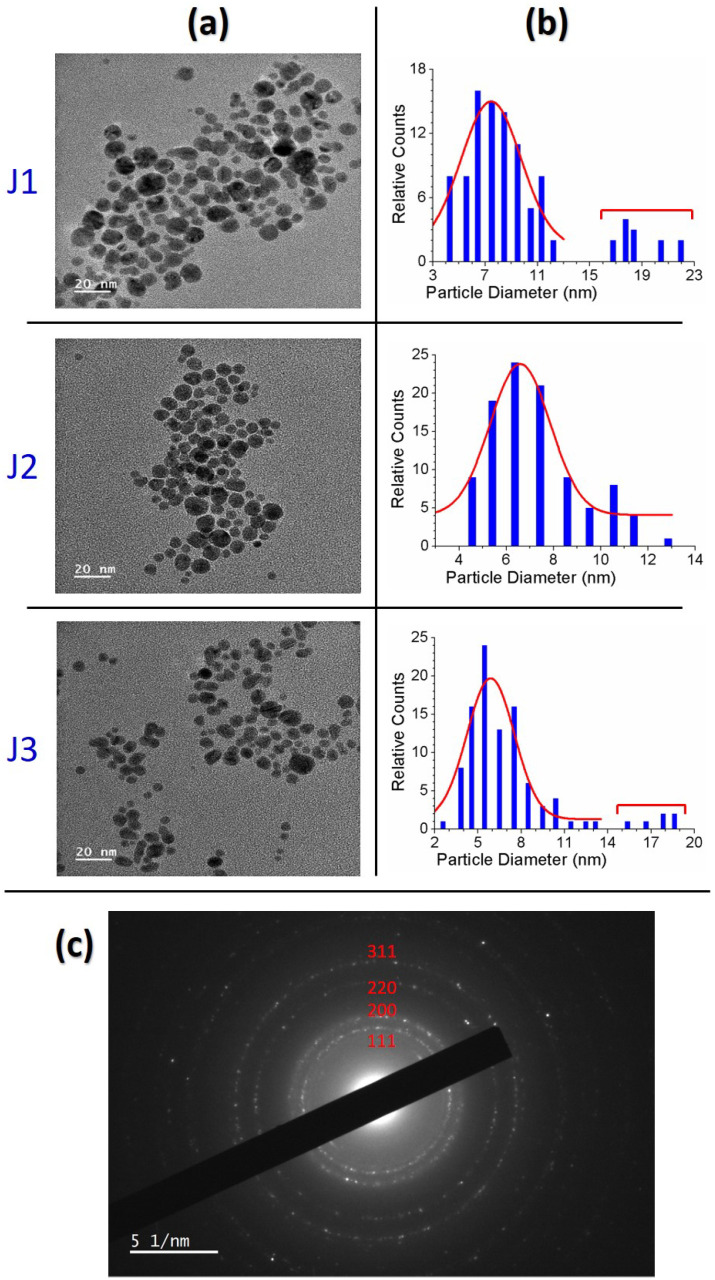
(**a**) HR-TEM images and (**b**) particle size distributions of the *Ag@Cit NPs* synthesized by treating $AgNO_{3}$-TSC-DW for 10 min with the J1, J2, and J3 jets. (**c**) Electron diffraction patterns of the *Ag@Cit NPs* synthesized with the J2 jet.

Figure 9a shows the HR-TEM images of the *Ag@Cit NPs* synthesized by treating $AgNO_{3}$-TSC-DW for 10 min with the J1, J2, and J3 jets. The *Ag@Cit NPs* by the J2 jet exhibited a single size distribution that could fit to Gaussian and Lorentzian functions, but the *Ag@Cit NPs* by the J1 and J3 jets caused two size distributions: one smaller and one larger. Considering that the *Ag@Cit NPs* by the J1, J2, and J3 jets exhibited a single SPR peak at approximately 406 nm, larger nanoparticles might be produced from the aggregation of nanoparticles during the drop casting of nanoparticles on the TEM grid. In the HR-TEM images of the *Ag@Cit NPs* by the J1 and J3 jets, smaller nanoparticles correspond to plasma-synthesized *Ag@Cit NPs*. Plasma-synthesized *Ag@Cit NPs* featured a quasi-spherical shape with average particle diameters of 7.5 ± 2.1, 6.6 ± 2.0, and 5.9 ± 2.1 nm for the J1, J2, and J3 jets, respectively, (Figure 9b). Figure 9c shows the electron diffraction patterns of the dense *Ag@Cit NPs*. The electron diffraction patterns of the *Ag@Cit NPs* are consistent with the {111}, {200}, {220}, and {311} crystallographic planes of face-centered cubic silver crystals. These results indicate that crystalline silver nanoparticles were produced by plasma treatment of $AgNO_{3}$-TSC-DW.

### 3.4. Plasma Synthesis Routes of Silver Nanoparticles

The plasma reduction routes of silver ion ($Ag^{+}$) to zero-valent silver ($Ag^{0}$) nanoparticles are well established, and are based on the two following reactions [5,23,43]:

(1) $$\begin{array}{ccc} & & Ag^{+}+e_{aq}^{-}⇌Ag^{0}, \end{array}$$

(2) $$\begin{array}{ccc} & & Ag^{+}+H⇌Ag^{0}+H^{+}. \end{array}$$

The reaction in Equation (1) is the reduction of silver ions by plasma electrons that are solvated into the aqueous solution. The main source of plasma electrons to reduce silver ions to *AgNPs* is the excited argon atoms. Plasma-excited argon atoms can provide plasma electrons that are required to reduce silver ions to *AgNPs*. The standard reduction potential of the silver ion is 0.80 V, which can instantly capture plasma electrons to form *AgNPs*. Equation (2) shows the reduction of silver ions through hydrogen atoms formed in the aqueous solution due to plasma–liquid interactions. Meanwhile, a large amount of hydrogen ions can digest silver nanoparticles (Equation (2)), leading to a smaller amount of *AgNPs*, as shown in Figure 8d.

Plasma consists of charged particles (electrons and ions), neutral particles, and UV light. When the plasma jet enters the aqueous solution, plasma radicals in gas phase can be transported to solution, and these solvated reactive species change the physicochemical properties of plasma-treated water. In the plasma-treated-DW and $AgNO_{3}$-TSC-DW, additional reactive species can be generated through the following plasma–liquid interactions [44,45,46,47,48,49]:

(3) $$\begin{array}{ccc} & & Ar+e^{-}\to Ar^{*}+e^{-}, \end{array}$$

(4) $$\begin{array}{ccc} & & M^{*}+N_{2}\to M+N+N, \end{array}$$

(5) $$\begin{array}{ccc} & & M^{*}+O_{2}\to M+O+O, \end{array}$$

(6) $$\begin{array}{ccc} & & M^{*}+H_{2}O\to M+H^{+}+OH+e_{aq}^{-}, \end{array}$$

(7) $$\begin{array}{ccc} & & e^{-*}+H_{2}O\to H^{+}+OH^{-}+e_{aq}^{-}, \end{array}$$

(8) $$\begin{array}{ccc} & & e^{-}+O_{2}\to O_{2}^{-}, \end{array}$$

(9) $$\begin{array}{ccc} & & H^{+}+O_{2}^{-}⇌HO_{2},\;pK_{a}=4.8, \end{array}$$

(10) $$\begin{array}{ccc} & & HO_{2}+HO_{2}\to H_{2}O_{2}+O_{2}. \end{array}$$

(11) $$\begin{array}{ccc} & & H^{+}+e_{aq}^{-}\to H, \end{array}$$

(12) $$\begin{array}{ccc} & & N+O\to NO, \end{array}$$

(13) $$\begin{array}{ccc} & & NO+O\to NO_{2}, \end{array}$$

(14) $$\begin{array}{ccc} & & NO+NO_{2}⇌N_{2}O_{3}, \end{array}$$

(15) $$\begin{array}{ccc} & & N_{2}O_{3}+H_{2}O⇌2HNO_{2}, \end{array}$$

(16) $$\begin{array}{ccc} & & 4NO+O_{2}+2H_{2}O\to 4NO_{2}^{-}+4H^{+}, \end{array}$$

(17) $$\begin{array}{ccc} & & H^{+}+NO_{2}^{-}⇌HNO_{2},\;pK_{a}=3.3, \end{array}$$

(18) $$\begin{array}{ccc} & & NO+O_{2}^{-}⇌NO_{3}^{-}, \end{array}$$

(19) $$\begin{array}{ccc} & & H^{+}+NO_{3}^{-}⇌HNO_{3},\;pK_{a}=-1.4, \end{array}$$

Here, *e* and $e_{aq}^{-}$ are dry (non-hydrated) and aqua (hydrated) electrons, respectively, and $M^{*}$ is any plasma ion with an ionization energy above the $H_{2}O$ ionization threshold. In the plasma-treated DW, hydrogen ions were generated through plasma-induced UV photolysis (Equation (6)), and/or electrolysis (Equation (7)) of water molecules, and then the formation of the nitrate (nitrite)–hydrogen ion complex results in acidic water (Equations (17) and (19)), as shown in Figure 7a. For plasma treatment of the TSC-DW, plasma-generated hydrogen ions were captured by citrate (Figure 7c). Similarly to the plasma-treated TSC-DW, the plasma-treated $AgNO_{3}$-TSC-DW exhibited higher pH values (Figure 7b).

Now we demonstrate the effects of citrate on the silver nanoparticle synthesis. Under higher pH conditions, *Ag@Cit NPs* can be efficiently synthesized from $AgNO_{3}$ and TSC ($Na_{3}C_{6}H_{5}O_{7}=Na_{3}Cit$) [50,51]:

(20) $$\begin{array}{ccc} & & 4Ag^{+}+Na_{3}Cit+2H_{2}O\to 4Ag^{0}+H_{3}Cit+3Na^{+}+H^{+}+O_{2}, \end{array}$$

(21) $$\begin{array}{ccc} & & H_{3}Cit⇌H_{2}Cit^{-}+H^{+},\;pK_{a}=3.13, \end{array}$$

(22) $$\begin{array}{ccc} & & H_{2}Cit^{-}⇌HCit^{2-}+H^{+},\;pK_{a}=4.76, \end{array}$$

(23) $$\begin{array}{ccc} & & HCit^{2-}⇌Cit^{3-}+H^{+},\;pK_{a}=6.40. \end{array}$$

Here, $Cit^{3-}=$${\left(C_{6}H_{5}O_{7}\right)}^{3-}$ is the citrate trianion. Citric acid ($C_{6}H_{8}O_{7}\;=\;H_{3}Cit$) can be oxidized to citrate, and the oxidation products of citric acid depend on pH [52]. Under neutral or basic conditions (6 ≤ pH ≤ 11), the dominant structure of oxidized citric acid is a citrate trianion ($C_{6}H_{5}O_{7}=Cit^{3-}$). In the plasma-treated $AgNO_{3}$-TSC-DW, plasma-generated aqua electrons convert hydrogen ions to hydrogen atoms (Equation (11)). Hydrogen-induced reduction of silver ions is the dominant process for the plasma synthesis of *Ag@Cit NPs* because the reduction potential of the hydrogen ion is lower than that of the silver ion. In the plasma-treated $AgNO_{3}$-TSC-DW, silver nanopartcles were produced from the reduction of silver ions through hydrogen atoms, and then a silver nanoparticle–citrate complex (*Ag@Cit NPs*) was formed [13].

(24) $$\begin{array}{ccc} & & 2Ag^{+}+2H+Cit^{3-}\to 2Ag^{0}+2H^{+}+Cit^{3-}, \end{array}$$

(25) $$\begin{array}{ccc} & & 2Ag^{0}+2H^{+}+Cit^{3-}\to {\left(Ag_{2}^{0}\cdot \cdot \cdot H_{2}Cit\right)}^{-}. \end{array}$$

Next, we consider the effects of plasma plume length on the plasma-synthesized silver nanoparticles. Experimentally, plasma treatment of the $AgNO_{3}$-DW did not produce a sufficient amount of silver nanoparticles, whereas that of $AgNO_{3}$-TSC-DW did. These results indicate that plasma electron-induced reduction of silver ions by reaction Equation (1) is not main synthesis route of silver nanoparticles under our experimental conditions. Next, citrate reduction of $AgNO_{3}$-TSC-DW without boiling or plasma treatment failed to produce a sufficient amount of silver nanoparticles. Significant amounts of silver nanoparticles could be synthesized only when the plasma jet was applied to $AgNO_{3}$-TSC-DW. Plasma-assisted citrate reduction of silver ions is more effective than citrate reduction of silver ions without plasma. Considering that reduction of silver ions by plasma electrons in the $AgNO_{3}$-DW did not produce a sufficient amount of silver nanoparticles, the reduction of silver ions in $AgNO_{3}$-TSC-DW is attributed to hydrogen atoms from the citrate (Equations (11) and (20)–(23)). In the plasma-treated $AgNO_{3}$-TSC-DW, hydrogen-induced reduction of silver ions is the main synthesis route of silver nanoparticles. Efficient synthesis of silver nanoparticles can be achieved by citrate reduction of silver salt under neutral or basic conditions (6 ≤ pH ≤ 11) (Equations (21)–(25)) [51,53]. The synergetic effect between citrate and plasma electrons plays an important role in the plasma-synthesis of silver nanoparticles. The amount of plasma-synthesized *Ag@Cit NPs* was determined by the hydrogen atoms that were produced from the plasma electron-induced reduction of hydrogen ions. As shown in Figure 8c, longer-duration plasma treatment produced a larger amount of *Ag@Cit NPs*, because prolonged plasma treatment of $AgNO_{3}$-TSC-DW led to more plasma electrons to be used for synthesis of *Ag@Cit NPs*. Regarding the effect of plasma plume length on the synthesis of silver nanoparticles, the amount of plasma-synthesized *Ag@Cit NPs* was largest in the J2 jet (Figure 8a). As shown in Figure 5b, the electrical conductivity was higher in the longer jets than in the J1 jet. The electrical conductivity of the J2 jet was significantly higher than that of the J1 jet, and the electrical conductivity of the J3 jet was slightly higher than that of the J2 jet. The longer jet produced higher electrical conductivity, indicating that more plasma electrons were generated in the longer jet (Figure 6). Meanwhile, the pH value of the J2 jet was higher than those of the shorter and longer jets, as shown in Figure 7b. That is, the J2 jet led to the highest pH value of the $AgNO_{3}$-TSC-DW.

To further study the effect of plasma plume length on the synthesis of *Ag@Cit NPs*, we consider the relationship between the amount of plasma-synthesized silver nanoparticles and the acidity of the plasma-treated medium. From the silver nanoparticle synthesis reaction (Equation (2)) for reduction of silver ions through hydrogen atoms,

(26) $$\begin{array}{ccc} & & \frac{\left[Ag^{0}\right]\;\left[H^{+}\right]}{\left[Ag^{+}\right]\;\left[H\right]}=K_{eq},\;\mathrm{at}\;\mathrm{equilibrium}. \end{array}$$

Here, $K_{eq}$ is the equilibrium constant of the reaction (Equation (2)) for silver nanoparticle synthesis. $\left[Ag^{0}\right]$ and $\left[Ag^{+}\right]$ are the concentrations of zero-valent silver nanoparticles and silver ions, respectively. $\left[H^{+}\right]$ and [H] are the concentrations of hydrogen ions and hydrogen atoms, respectively. At a fixed concentration of silver ions, a higher hydrogen atom concentration can produce a higher silver nanoparticle concentration, but a higher hydrogen ion concentration can digest silver nanoparticles. In the J1 and J3 jets, a lower pH indicates a larger number of hydrogen ions, which can convert *AgNPs* to silver ions, leading to a smaller number of *Ag@Cit NPs*. To summarize, the largest amount of *Ag@Cit NPs* obtained in the J2 jet is attributed to a higher pH and a larger number of aqua electrons. Plasma-assisted citrate reduction of silver ions is the main synthesis route of *Ag@Cit NPs*. In addition, citrate facilitates the conversion of silver ions to *Ag@Cit NPs* and stabilizes *Ag@Cit NPs* in an aqueous solution. Plasma-synthesized *Ag@Cit NPs* are well-dispersed in an aqueous solution. The formation of stabilized nanoparticles is due to electrostatic repulsion of citrate-capped *AgNPs*, as confirmed by the zeta potential of −55 mV.

### 3.5. Degradation Activity of Methylene Blue Dye Treated by Plasma-Synthesized Silver Nanoparticles

**Figure 10:**
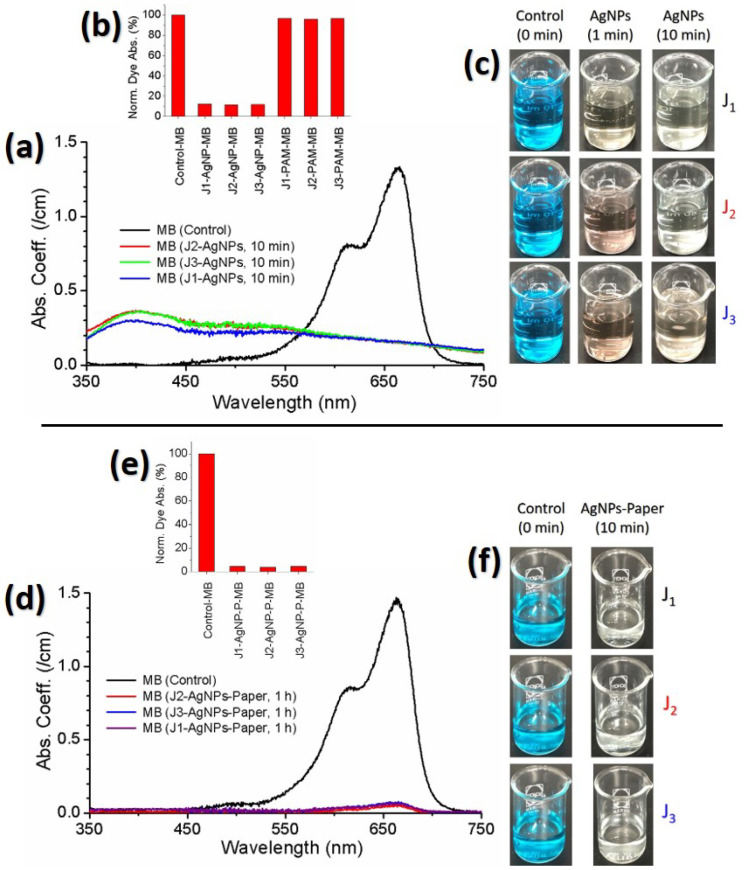
(**a**) Absorption spectra of *Ag@Cit NP*-treated MB solutions. (**b**) Dye degradation effects of *Ag@Cit NPs* and plasma-activated media (PAM). (**c**) Photos of *Ag@Cit NP*-treated MB. (**d**) Absorption spectra of *Ag@Cit NP*-paper-treated MB solutions. (**e**) Dye degradation effects of *Ag@Cit NP*-paper. (**f**) Photos of *Ag@Cit NP*-paper-treated MB. *Ag@Cit NPs* were synthesized by treating $AgNO_{3}$-TSC-DW for 10 min with the J1, J2, and J3 jets. Nanoparticle-free plasma-activated media were prepared by treating TSC-DW for 10 min with the J1, J2, and J3 jets. Solutions with 26 μM MB were treated by plasma-synthesized *Ag@Cit NPs*.

To confirm potential applications of the *Ag@Cit NPs* for the degradation of environmental pollutants, we investigated the degradation activity of plasma-synthesized *Ag@Cit NPs* on MB dye. The degradation activity of *Ag@Cit NP* treatment on MB was measured by optical absorption spectroscopy [54]. Figure 10a exhibits the absorption spectra of *Ag@Cit NP*-treated and -untreated MB solutions. The corresponding photos of *Ag@Cit NP*-treated MB solutions are displayed in Figure 10c. The pristine MB solution exhibited a broad absorption band with a strong peak at 670 nm and a shoulder at 615 nm [55]. The 670 nm absorption can be assigned to the MB monomer, and the 615 nm absorption can be attributed to the MB dimer. Comparing the *Ag@Cit NP*-treated MB with the pristine MB, the 670nm absorption peak of MB disappered by the *Ag@Cit NP* treatment. However, the *Ag@Cit NP*-treated MB exhibited significant absorption at 670 nm. To confirm the degradation activity of *Ag@Cit NPs* on MB, we measured the degradation activity of the nanoparticle-free plasma-activated medium (PAM). As positive controls of the plasma-synthesized *Ag@Cit NPs*, the TSC-DW solutions were treated with the J1, J2, and J3 jets for 10 min. As shown in Figure 10b, the 670 nm absorption intensity of PAM-treated-MB was similar to that of pristine MB, indicating that the degradation activity of *Ag@Cit NPs* is stronger than that of PAM. The nonzero absorption of *Ag@Cit NP*-treated MB at 670 nm may be ascribed to dye degradation products induced from the interaction of MB with *Ag@Cit NPs*. One possible explanation for the formation of dye degradation products by the *Ag@Cit NP* treatment is AgNP-induced electron transfer from $BH_{4}^{-}$ ions to MB dye [56]. To fully understand the dye degradation mechanism by the *Ag@Cit NP* treatment, it will be necessary to conduct a more detailed study for exploring the dye degradation performance of *Ag@Cit NPs* and plasma-activated medium.

Figure 10d exhibits the absorption spectra of *Ag@Cit NP*-paper-treated MB solutions. The corresponding photos of *Ag@Cit NP*-paper-treated MB solutions are displayed in Figure 10f. Comparing the 670 nm absorption peak of *Ag@Cit NP*-paper-treated MB with that of pristine MB, the 670 nm absorption peak of *Ag@Cit NP*-paper-treated MB decreased to almost zero, as shown in Figure 10e. Absorption spectra of the *Ag@Cit NP*-paper-treated MB solutions do not have an SPR absorption band of *Ag@Cit NPs*, indicating that degradation of MB dye without remnant silver nanoparticles was achieved by the *Ag@Cit NP*-paper treatment.

## 4. Conclusions

Citrate-capped silver nanoparticles were synthesized by treating $AgNO_{3}$-TSC-DW with atmospheric-pressure argon plasma jets. *Ag@Cit NPs* with an average particle diameter of about 5.9−7.5 nm were obtained after plasma treatment for 10 min. Plasma-synthesized *Ag@Cit NPs* have a face-centered cubic structure, and their absorption spectra exhibited surface plasmon resonance peaks at approximately 406 nm. Citrate facilitated the conversion of silver ions to *Ag@Cit NPs* and stabilized *AgNPs* in an aqueous solution. Plasma-synthesized *Ag@Cit NPs* were well-dispersed in the aqueous solution. The amount of *Ag@Cit NPs* increased in a plasma-treatment duration-dependent manner. The acidity and electrical conductivity measurements showed that a larger amount of *Ag@Cit NPs* were produced from the 8.5 cm plume jet with a higher pH and a larger number of aqua electrons. Plasma synthesis of *Ag@Cit NPs* was more effective in the 8.5 cm plume jet than in the shorter and longer plume jets. These results indicate that the synergetic effect between plasma electrons and citrate played an important role in the plasma synthesis of *Ag@Cit NPs*. Plasma-synthesized *Ag@Cit NPs* exhibited significant dye degradation activity for methylene blue.

## Data Availability

Not applicable.
